# FKBP51 Affects TNF-Related Apoptosis Inducing Ligand Response in Melanoma

**DOI:** 10.3389/fcell.2021.718947

**Published:** 2021-09-13

**Authors:** Martina Tufano, Elena Cesaro, Rosanna Martinelli, Roberto Pacelli, Simona Romano, Maria Fiammetta Romano

**Affiliations:** ^1^Dipartimento di Medicina Molecolaree Biotecnologie Mediche, Università degli Studi di Napoli Federico II, Naples, Italy; ^2^Dipartimento di Medicina, Chirurgia ed Odontoiatria, Università degli Studi di Salerno, Baronissi, Italy; ^3^Dipartimento di Scienze Biomediche Avanzate, Università degli Studi di Napoli Federico II, Naples, Italy

**Keywords:** TRAIL, melanoma, cell death, FKBP51, YY1

## Abstract

Melanoma is one of the most immunogenic tumors and has the highest potential to elicit specific adaptive antitumor immune responses. Immune cells induce apoptosis of cancer cells either by soluble factors or by triggering cell-death pathways. Melanoma cells exploit multiple mechanisms to escape immune system tumoricidal control. FKBP51 is a relevant pro-oncogenic factor of melanoma cells supporting NF-κB-mediated resistance and cancer stemness/invasion epigenetic programs. Herein, we show that FKBP51-silencing increases TNF-related apoptosis-inducing ligand (TRAIL)-R2 (DR5) expression and sensitizes melanoma cells to TRAIL-induced apoptosis. Consistent with the general increase in histone deacetylases, as by the proteomic profile, the immune precipitation assay showed decreased acetyl-Yin Yang 1 (YY1) after FKBP51 depletion, suggesting an impaired repressor activity of this transcription factor. ChIP assay supported this hypothesis. Compared with non-silenced cells, a reduced acetyl-YY1 was found on the DR5 promoter, resulting in increased DR5 transcript levels. Using Crispr/Cas9 knockout (KO) melanoma cells, we confirmed the negative regulation of DR5 by FKBP51. We also show that KO cells displayed reduced levels of acetyl-EP300 responsible for YY1 acetylation, along with reduced acetyl-YY1. Reconstituting FKBP51 levels contrasted the effects of KO on DR5, acetyl-YY1, and acetyl-EP300 levels. In conclusion, our finding shows that FKBP51 reduces DR5 expression at the transcriptional level by promoting YY1 repressor activity. Our study supports the conclusion that targeting FKBP51 increases the expression level of DR5 and sensitivity to TRAIL-induced cell death, which can improve the tumoricidal action of immune cells.

## Introduction

Melanoma is insidious cancer that is highly resistant to cytotoxic treatments. This neoplasia is among the most immunogenic tumors and has the highest potential to elicit specific adaptive antitumor immune responses (Passarelli et al., 2017). The brilliant results obtained with immunotherapy have dramatically changed the therapeutic outcomes of advanced melanomas (Luke et al., 2017). However, the frequency of non-responders to immune-based treatment remains high. Therefore, more studies are needed to understand melanoma escape from immune surveillance. Immune cells induce apoptosis of cancer cells either by soluble factors, i.e., perforin and granzymes, or triggering cell-death pathways (Marzagalli et al., 2019). TNF-related apoptosis-inducing ligand (TRAIL), on NK and CD8 T lymphocytes, plays an essential role in controlling tumor immune surveillance (Passarelli et al., 2017). TRAIL is a TNF family member protein considered ideal cancer therapeutic because of its selective cytotoxicity against malignancies (Walczak and Krammer, 2000). Among factors regulating melanoma sensitivity to TRAIL, the level of cell-surface expression of specific death receptors (DR), in particular DR5, plays a relevant role in TRAIL response, as suggested by several studies (Zhang et al., 1999; Hersey and Zhang, 2001; Nguyen et al., 2001). For this reason, agents to increase the cell-surface expression of TRAIL-DRs on melanoma cells are expected to improve the clinical efficacy of soluble TRAIL (Chen et al., 2007). According to the Human Protein Atlas,^1^ expression of DR5 is moderate, as of intensity, but broad (>75% of melanoma cells) in most tumor tissues, with a localization either cytoplasmic and membraneous. Although not referred to as a prognostic factor of melanoma, DR5 expression positively correlates with the overall survival of melanoma patients (see text footnote 1).

Melanoma cells exploit multiple mechanisms to escape immune system tumoricidal control (Eddy and Chen, 2020). Our laboratory has found that the immunophilin FKBP51 is a relevant pro-oncogenic factor of melanoma cells (Romano et al., 2010, 2013). Targeting FKBP51 produced an increase in melanoma cell apoptosis induced by various cytotoxic stimuli (Romano et al., 2010, 2015). In an attempt to discover and characterize molecules and pathways involved in the increased sensitivity to cell death of FKBP51-silenced melanoma cells, we performed a preliminary study of the protein expression profiles in melanoma cells. To this end, cells were either depleted or not in FKBP51, with ionizing radiation (IR) serving as cell death stimulus. Ingenuity pathway analysis (IPA) of the proteome highlighted “cell death and survival” pathway as the first scored map based on the enrichment distribution sorted by statistically significant map set. Along with tumor necrosis factor/receptor superfamily members, epigenetic modulators, including several histone deacetylases (HDACs), were positively regulated by FKBP51 silencing. DR5 expression is held at the transcriptional level by Yin Yang 1 (YY1). Acetylation of YY1 by EP300 confers the transcriptional repressor activity, deacetylation by HDAC1 and HDAC2 contrasts such repressor activity (Yao et al., 2001). We investigated FKBP51 regulation of DR5 expression and sensitivity to TRAIL of melanoma cells, and the involvement of acetyl-YY1 in such regulation. Using two different melanoma cell lines and three different methods for FKBP51 modulation, namely short interfering RNA (siRNA), short hairpin RNA (sh-RNA), and CRISPR/Cas9 KO, we show that FKBP51 silencing increased DR5 expression and enhanced sensitivity of melanoma cells to TRAIL-induced apoptosis. The mechanism of DR5 regulation involved the repressor activity of YY1 that was attenuated by FKBP51 silencing.

## Materials and Methods

### Cell Cultures, Transfection, and Reagents

Human melanoma cell lines SAN and A375 were obtained and cultured as described previously (Romano et al., 2010). For FKBP51 knockin, knockdown, and knockout (KO), cells were transfected using the K2 Transfection System (Biontex, Munich, Germany), in accordance with the manufacturer’s recommendations, as previously described (D’Arrigo et al., 2017). Briefly, 24 h before transfection cells were seeded into six-well plates at a concentration of 4 × 10^5^ cells/ml to obtain confluency of 60–70%. FKBP51 silencing was performed using short-interfering oligoribonucleotides. The siFKBP51 and the control non-silencing RNA (NSRNA) were from Qiagen (Valencia, CA, United States). The A375 cell line stably knocked down for FKBP51 (shFKBP51.3) was obtained as previously described (Romano et al., 2015). For overexpressing FKBP51, a True-ORF-Myc-DDK-tagged expression was used (OriGene Technologies, Rockville, MD, United States), which carried the cDNA of the human FKBP5. Control cells were transfected with the related empty vector (EV). For the ectopic expression of FKBP51 mutants, a True-ORF-Myc-DDK-tagged expression carrying the cDNA of the human FKBP5 transcript variant 4 was used (OriGene Technologies), along with plasmids encoding pRK5 Flag-tagged FKBP51 harboring TPR mutation (Flag-FKBP51-mutTPR; K352A/R356A) and PPIase mutation (Flag-FKBP51-mutPPIase; FD67DV) that were kindly provided by Theo Rein (Max Planck Institute of Psychiatry, Munich, Germany) (Romano et al., 2015). For the establishment of the A375 knockout cell line, A375 cells were transfected with a CRISPR/Cas9 KO plasmid along with an HDR plasmid for the puromycin resistance (Santa Cruz Biotechnology, CA, United States). FKBP51 HDR plasmid alone was transfected to generate control cells. Then, to establish a stable KO cell line, after 24 h from transfection, cells were selected with 200 ng/ml puromycin (Merck, Darmstadt, Germany), while single FKBP51-KO clones were obtained by a limiting dilution of transfected cells grown in puromycin. For IR experiments, at 48 h after transfection, cells were irradiated with a 6MV X-ray of a linear accelerator (Primus, Siemens, München, Deutschland) (Romano et al., 2010) and processed according to the different experimental procedures. Treatment with thricostatin (TSA; Merck) and TRAIL (ImmunoTools, Friesoythe, Germany) was performed after 6 h from transfection and used at the concentrations indicated in the section “Results.”

### Antibody Array

SAN melanoma cells were transfected as described in the paragraph “cell cultures, transfection, and reagents” with a specific small interfering (si) RNA for FKBP51 or a non-silencing (NS) RNA as a control. After 24 h from transfection, cells were irradiated with 6MV X-ray of the linear accelerator at the dose of 4 Gy. Six hours later, cells were harvested for protein extraction. Lysates were extracted in RIPA-modified buffer (Romano et al., 2013), and each experimental point was labeled with a different cyanine (Cy3 or Cy5) and used for the XPRESS Panorama Antibody Array Kit (Sigma-Aldrich, Saint Louis, MO, United States). This antibody array contains 725 different antibodies each spotted in duplicate on nitrocellulose-coated glass slides useful to detect a wide variety of proteins, which can be categorized in seven subgroups, including apoptotic, cell cycle, nuclear signaling, stress, calcium-associated, cytoskeleton, and signal transduction based on biological functions.^2^ Bioinformatic analysis of differentially expressed protein in FKBP51-silenced melanoma cells was performed through IPA software as previously described by Pisanti et al. (2021). Briefly, outcomes from protein microarray analysis were uploaded into the IPA system of Qiagen for core analysis using the ingenuity pathway knowledge base (IPKB). IPA was performed to identify canonical pathways, diseases, and functions and gene networks and to categorize differentially expressed genes in specific diseases and functions. A right-tailed Fisher’s exact test was used to calculate a *p*-value determining the probability that each biological function and/or disease assigned to that data set is due to chance alone.

### Western Blot and Immunoprecipitation

Whole lysates were obtained through the homogenization of cell pellet in modified RIPA buffer (Romano et al., 2015) and assayed by immunoblot as previously described (Romano et al., 2015). The primary antibodies against YY1 (mouse monoclonal, Cat# H00007528-M01, RRID:AB_464157, Abnova, Taipei City, Taiwan), antiacetyl-lysine (rabbit polyclonal, Cat# PAB10348, RRID:AB_1671598, Abnova), anti-DR5 (rabbit polyclonal, Cat# 3696, RRID:AB_10692107, Cell Signaling, Danvers, MA, United States), antiacetyl CBP/EP300 (rabbit polyclonal, Cat# 4771, RRID:AB_2262406, Cell Signaling), anti-CBP/EP300 (mouse monoclonal, Cat# NB 100-617, RRID:AB_525941, Novus Biological, CO., United States), anti-G3PDH (mouse monoclonal, Cat# sc-32233, RRID:AB_627679, Santa Cruz Biotechnology), and antivinculin (mouse monoclonal, Cat# sc-55465, RRID:AB_630433, Santa Cruz Biotechnology) were diluted 1:1,000; anti-M2-flag (mouse monoclonal, Cat# F3165, RRID:AB_259529, Merck) and anti-γ-tubulin (mouse monoclonal, Cat# T5326, RRID:AB_532292, Merck) were used 1:5,000; anti-FKBP51 (rabbit polyclonal, Cat# NB100-68240, RRID:AB_1108566, Novus Biologicals) was diluted 1:3,000; and anti-β-actin HRP conjugated (mouse monoclonal, Cat# A3854, RRID:AB_262011, Merck) was diluted 1:10,000. Secondary antibody HRP conjugated were purchased from Microtech [ImmunoReagents ant-imouse and anti-rabbit (Cat# GtxRb-003-DHRPX, RRID:AB_2884989), Microtech, Pozzuoli, Italy] and diluted 1:5,000. Signals were revealed thanks to the incubation of membranes with Western Blotting Luminol Reagent (Cat# sc-2048, RRID:AB_10188880, Santa Cruz Biotechnology). For YY1 immunoprecipitation, cells were treated as for ChIP analysis (see section “Chromatin Immunoprecipitation”), and the lysates were immunoprecipitated with anti-YY1 antibody on a shaker at 4°C overnight. On the next day, the immunoprecipitated proteins were collected by incubation with protein A/G plus agarose beads (Cat# sc-2003, RRID:AB_10201400, Santa Cruz Biotechnology) on a shaker at 4°C for 2 h, then beads were washed with cell lysis buffer and resolved by SDS-PAGE.

### Flow Cytometry

DR5 expression was assessed by immunofluorescence using an antihuman TRAIL-R2-phycoerythrin (PE)-conjugated antibody from R&D Systems (Cat# FAB6311P, RRID:AB_2204812, Minneapolis, MI, United States). A PE-conjugated control Ig isotype was used to assess non-specific binding. Cells were harvested and stained for 30 min in the dark at 4°C. After washing with PBS, cells were acquired and analyzed with BD FACScan Cytometer or BD Accuri^TM^ C6 Cytometer (BD, Becton Dickinson, NJ, United States). Apoptosis was assessed by propidium iodide (PI) incorporation (Romano et al., 2010) to measure the DNA content of permeabilized cells (hypodiploid cells). Briefly, after each specific treatment, cells were collected and washed twice with PBS, then cells were resuspended in 100 μl of buffer containing 10 μl PI solution (Romano et al., 2010). After 30 min of incubation in the dark at 4°C, cells were analyzed with BD FACScan.

### RT-qPCR

Total RNA was extracted from cells by TRIzol (Invitrogen, Carlsbad, CA, United States). Each RNA was used for cDNA synthesis with iScript Reverse Transcription (Bio-Rad, Hercules, CA, United States). Relative gene expression was quantified by qPCR with 2^–ΔΔCt^ comparative method using the SsoAdvanced^TM^ SYBR Green Supermix (Bio-Rad) and specific qPCR primers. Oligo primers used for DR5 and FKBP51 were purchased from Qiagen (validated QuantiTect primers, KA, United States) and run along with coamplified housekeeping genes β-actin and 18S whose sequences were previously reported (Romano et al., 2015).

### Chromatin Immunoprecipitation

Chromatin immunoprecipitation was performed as previously described (Busiello et al., 2017). Briefly, A375 cells transfected with siRNAFKBP51 or scrambled, as control, were cross-linked with HCHO. The fixed chromatin was immunoprecipitated overnight with 1 μg anti-YY1 antibody (mouse monoclonal, Cat# H00007528-M01, RRID:AB_464157, Abnova) or IgG (Merck), as control of immunoprecipitation reaction. The immunocomplexes were recovered by protein A/G plus Agarose (Santa Cruz Biotechnology), and the crosslinking was reversed at 65°C overnight. Subsequently, the DNA was recovered by phenol/chloroform extraction and ethanol precipitation. The analysis of immunoprecipitated DNA and input controls were performed in triplicate by quantitative real-time PCR using a Master Mix SYBRGreen (Bio-Rad). The amount of DNA immunoprecipitated by using YY1 antibody or IgG was calculated relative to the input DNA. One percent of starting chromatin was used as input DNA. Primer sequences were as follows: DR5 promoter region (Fw: ggaaggggagaagatcaagacg; Rev: tggtttgtttctgggtcctgtc); DR5 Intron 2 region (Fw: actgcgctgggtccaaaattc; Rev: acgccactatacccagctaatg); and unrelated GAPDH region (UNR) (Fw: aggtcatccatgacaactttgg; Rev: ttgtcataccaggaaatgagct). For YY1 acetylation analysis, the beads with bound immunocomplexes were resuspended in Laemmli buffer and boiled at 95°C for 10 min. Finally, the supernatant was analyzed by Western blot (see section “Western Blot and Immunoprecipitation”).

### Statistical Analysis

Calculation of means and standard deviations was performed with Microsoft Excel. The Student’s *t*-test and ANOVA’s multiple comparisons test were used to analyze the differences between the values from two groups and multiple groups, respectively. *p*-Value ≤ 0.05 was considered statistically significant.

## Results

### Increased DR5 Expression and TRAIL Sensitivity in FKBP51-Depleted Melanoma Cells

**FIGURE 1:**
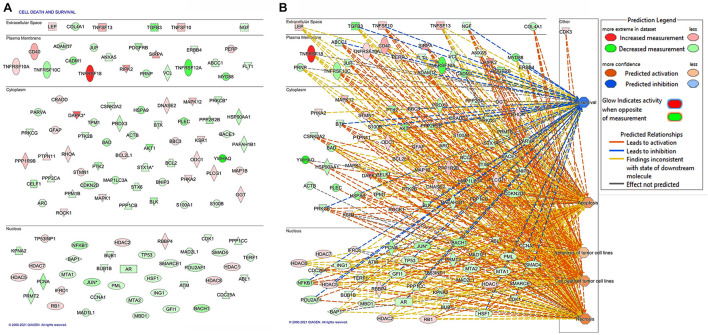
Bioinformatic analysis of differentially expressed protein in FKBP51-silenced melanoma cells. **(A)** The 117 differentially expressed proteins in FKBP51-silenced melanoma cells associated with cell death and survival. **(B)** Gene networks generated by IPA pathway analysis from the 117 differentially expressed proteins. Upregulated proteins are shown in red; the downregulated ones are in green. The brightness of color is related to the fold change, and the darker the color, the higher the fold change.

Protein expression profiles in melanoma cells that were either depleted or not in FKBP51 are illustrated in Figure 1 and Supplementary Figure 1. IPA was used to categorize differentially expressed proteins in FKBP51-silenced melanoma cells to diseases and functions (Figure 1 and Supplementary Figure 1). FKBP51 silencing significantly affects many cellular functions (Supplementary Figure 1), including cell death and survival (*p*−value 5.36 × 10^–54^, 117 affected proteins), cellular development (*p*−value 9.46 × 10^–41^, 113 affected proteins), cellular growth and differentiation (*p*−value 9.46 × 10^–41^, 112 affected proteins), cell cycle (*p*−value 1.64 × 10^–39^, 84 affected proteins), and cancer (*p*−value 1.9 × 10^–32^, 138 affected proteins). Figure 1 illustrates differentially expressed proteins in FKBP51-silenced melanoma cells (Figure 1A), along with IPA-generated networks (Figure 1B).

**FIGURE 2:**
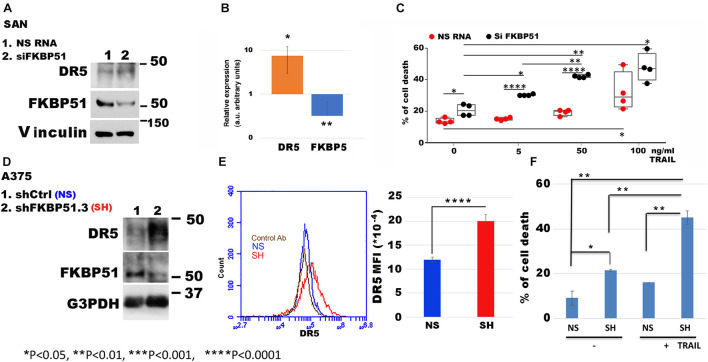
FKBP51 targeting affects DR5 expression and TNF-related apoptosis-inducing ligand (TRAIL) sensitivity to apoptosis. **(A)** Western blot assay of DR5 and FKBP51 expression in SAN melanoma cell line silenced or not for FKBP51. Vinculin was used as a loading control. The blot is representative of three experiments. **(B)** Analysis by RT-qPCR of DR5 and FKBP5 gene expression in SAN cell line in the condition of FKBP51 silencing (*N* = 5). Expression was relative to NSRNA-treated cell expression that was used as the reference sample (expression = 1). **(C)** Graphic representation of flow cytometric values of cell death in SAN melanoma cell cultures with TRAIL (each bullet represents the mean of triplicates). **(D)** Analysis by Western blot assay of DR5 and FKBP51 expression in A375 melanoma cells stably transfected with a non-silencing (NS) or FKBP51-silencing (SH) short hairpin RNA. G3PDH was used as a loading control. **(E)** Left, flow cytometric histograms in overlay of DR5 expression, measured in NS or SH A375 cells. Right, mean ± SD (*N* = 4) of DR5 mean fluorescence intensities. **(F)** Mean ± SD (*N* = 3) of cell death values in NS or SH A375 cell cultures with 100 ng/ml TRAIL.

It must be noted that IPA includes both DR4 and DR5 with the symbol TNFRSF10A. Because DR5 positively correlates with the overall survival of melanoma patients (see text footnote 1), we looked at role of FKBP51 in the regulation of DR5 expression in melanoma cells. Using the same SAN melanoma cell line (Romano et al., 2010) assessed for protein profiles, we confirmed that FKBP51 silencing produced DR5 upregulation by Western blot (Figure 2A) and RT-qPCR (Figure 2B). Moreover, a TRAIL dose-response assay (Figure 2C) showed that FKBP51-silenced melanoma cells had increased sensitivity to TRAIL. Cell death values (% mean ± StDev) were 13.5 ± 1.7, 15.0 ± 0.8, 18.7 ± 1.8, and 31.7 ± 12.2 for 0, 5, 50, and 100 ng/ml TRAIL, respectively (Figure 2C, red bullets, *N* = 4, means of triplicates). Melanoma cells treated with NSRNA appeared to be sensitive to 100 ng/ml TRAIL (Figure 2C). Differently, FKBP51 silencing induced sensitivity to TRAIL even at the lowest dose of 5 ng/ml. Values of FKBP51-silenced melanoma were 20.2 ± 3.7, 30.2 ± 0.5, 41.7 ± 0.9, and 47.2 ± 9.0 in 0, 5, 50, and 100 ng/ml TRAIL cultures, respectively (Figure 2C, black bullets, *N* = 4, means of triplicates). The effect of FKBP51 silencing on DR5 expression was confirmed by Western blot (Figure 2D) and flow cytometry (Figure 2E) in A375 melanoma cell line, which was stably silenced for FKBP51 (shFKBP51.3) with a sh-RNA, and generated as previously described (Romano et al., 2015). As shown in Figure 2F, ShFKBP51.3 cells were more sensitive than non-silenced cells (shCtrl) to apoptosis induced by 50 ng/ml TRAIL.

### HDAC Inhibitor Trichostatin a Counteracts DR5 Upregulation in FKBP51-Depleted Cells

**FIGURE 3:**
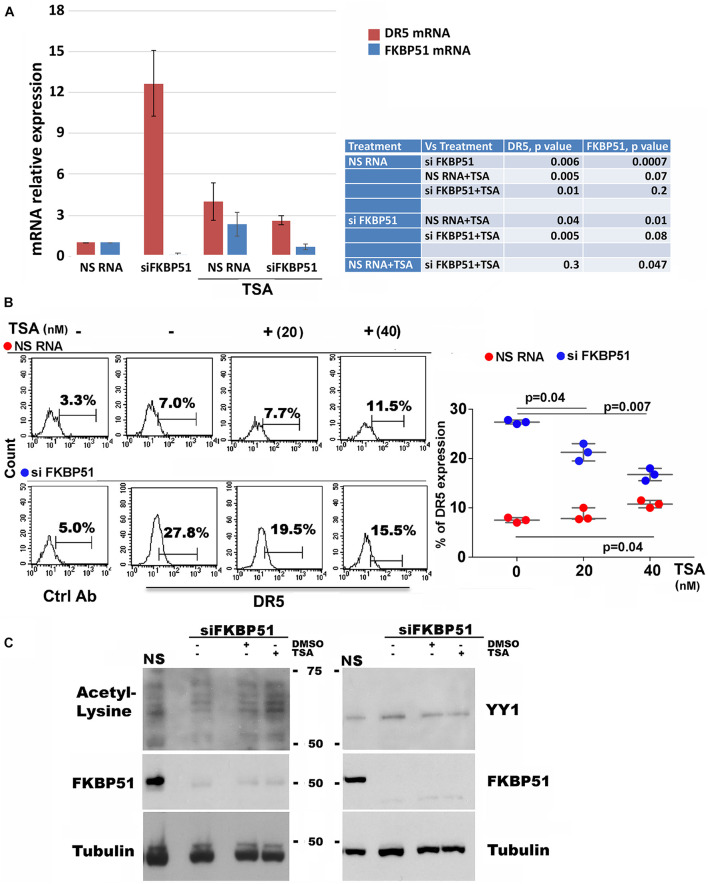
TSA counteracts the effect of FKBP51 silencing on DR5 expression. **(A)** Analysis by qPCR of DR5 mRNA (red column) and FKBP51 mRNA (blue column) expression in SAN cell line, silenced or not for FKBP51 and cultured with or without 40 nM TSA (*N* = 3). **(B)** Effect of TSA on DR5 expression on SAN melanoma cells silenced or not for FKBP51. Left, flow cytometric histograms of DR5 expression. Right, graphic representation of mean ± SD (*N* = 3) of DR5 (%) expression, in response to 20, and 40 nM TSA. **(C)** Western blot assay (representative of three different experiments) performed with antiacetyl lysine antibody shows a reduced acetylation pattern of FKBP51-silenced (SiFKBP51) A375, in comparison with non-silenced (NS)A375, which was contrasted by TSA.

Because, as by protein profiles (Figures 1B,C), several HDACs (HDAC-1, −2, −5, −7, and −8) appeared to be increased in FKBP51-silenced melanoma cell, we used trichostatin A (TSA) to investigate whether this agent modulated the effect of FKBP51 silencing on DR5 expression. Measurement of the transcript level of DR5 (Figure 3A, red column) showed that siFKBP51-induced upregulation of *DR5* gene expression contrasted by 40 nM TSA. The concomitant measure of FKBP51 mRNA (Figure 3A, blue column) confirmed the efficacy of siRNA in downmodulating FKBP51. Also, TSA slightly increased the expression of DR5 in NSRNA cells (Figure 3A). Analysis of protein expression by flow cytometry (Figure 3B) was in line with RT-qPCR data. TSA, indeed, slightly, but significantly, increased DR5 expression on the plasma membrane of NSRNA-treated cells (Figure 3B, red bullets) and contrasted the upregulation effect of siFKBP51 (Figure 3B, blue bullets). TSA inhibits HDAC1-HDAC2-mediated deacetylation of YY1, thus raising the hypothesis that the effect of TSA could involve this transcription factor. We, therefore, looked at the levels of YY1 and acetylated protein in FKBP51-silenced melanoma cells, cultured in the absence or presence of TSA. As shown in Figure 3C, compared with levels in non-silenced cells, acetylated protein appeared to be decreased in FKBP51-silenced cells. The addition of TSA, but not the vehicle DMSO, recovered the acetylated protein level. However, the level of YY1 did not substantially change in the same conditions.

### FKBP51 Affects the Binding of YY1 Repressor to DR5 Gene

**FIGURE 4:**
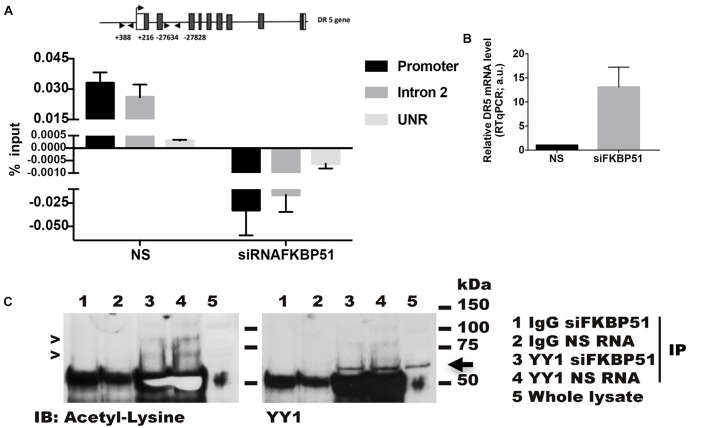
FKBP51 silencing affects Yin Yang 1 (YY1) binding on DR5 gene and its lysine acetylation status. **(A)** ChIP assay performed with an anti-YY1 antibody in A375 cells silenced for FKBP51 (siFKBP51) or control cells (NS). Quantitative RT-qPCR analysis was performed using primers covering YY1 putative binding sites in promoter and intron regions of the DR5 gene. An unrelated region (UNR) was used as a negative control. The histogram shows the amount of DNA immunoprecipitated by using YY1 antibody subtracting the amount of background IgG, both calculated relative to the input DNA (*N* = 3). **(B)** DR5 expression levels were analyzed by RT-qPCR (right panel). **(C)** YY1 was immunoprecipitated in siRNAFKBP51 and NS cells with anti-YY1 antibody or control immunoglobulin (IgG), as indicated. After immunoprecipitation, Western blot analysis was performed with antiacetyl lysine antibody (left panel). The arrows indicate acetylated YY1. The amount of immunoprecipitated YY1 was visualized by Western blot analysis with anti-YY1 antibody (right panel).

Because acetylation of YY1 is essential for its repressor activity and, as previous findings suggested a reduced acetylation pattern in protein extracted by FKBP51-silenced cells, we performed a chromatin immunoprecipitation assay (X-ChIP) to investigate whether FKBP51 influenced the YY1 binding to chromatin. The fixed chromatin was immunoprecipitated with an anti-YY1 antibody from A375 melanoma cells (silenced or not for FKBP51) and analyzed by RT-qPCR. Because, by *in silico* analysis, we found a putative YY1 binding motif in the second intron of DR5 gene, we analyzed the binding to the promoter region along with such a genomic region. In Figure 4A, a representative result from three different experiments shows that YY1 binds the promoter and the intronic region of DR5 in control cells and is detached from these binding sites in FKBP51-silenced cells. As expected, the decrease of YY1 chromatin binding was accompanied by an increase of DR5 transcription levels (Figure 4B). Furthermore, the immunoprecipitation of YY1 from protein extracted by A375 cells showed that acetyl-YY1 was reduced by FKBP51 downmodulation (Figure 4C, lane 3) in comparison with level in NSRNA cells (Figure 4C, lane 4). These results suggest that reduced YY1 DNA-binding activity in FKBP51-silenced cells corresponds to reduced YY1 acetylation.

### Ectopic FKBP51 Counteracts DR5 Upregulation in Crispr/Cas9-KO

**FIGURE 5:**
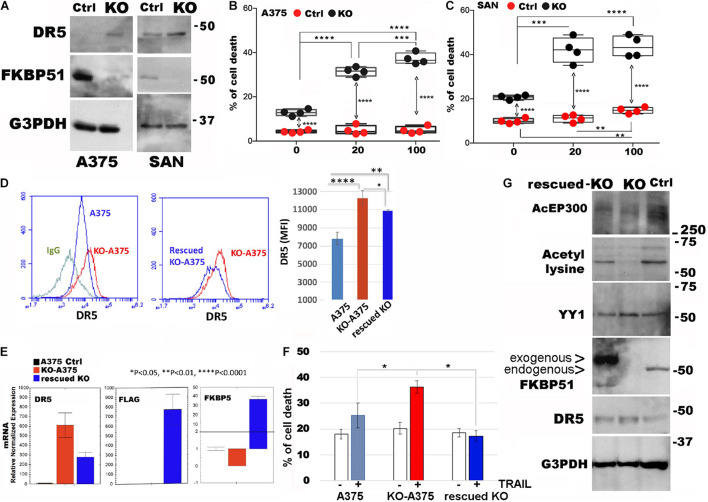
Ectopic FKBP51 counteracts DR5 upregulation in Crispr/Cas9-knockout (KO). **(A)** Western blot assay of DR5 levels in Crispr/Cas9 KO-A375, and KO-SAN cell lines. Graphic representation of flow cytometric values of cell death in A375 **(B)** and SAN **(C)** melanoma cell cultures with 20 and 100 ng/ml TRAIL (each bullet represents the mean of triplicates). **(D)** Left, flow cytometric histograms in overlay of DR5 expression, measured in A375 or KO-A375 or FKBP51-rescued KO-A375 cells. The histogram of KO-A375 moves to the right of that of wtA375, indicating increased expression. The histogram of rescued KO-A375 moves to the left of that of KO-A375, indicating reduced expression. Right, mean ± SD (*N* = 3) of DR5 mean fluorescence intensities (MFI). **(E)** Measurement by qPCR of DR5mRNA, Flag mRNA, and FKBP5 mRNA in the same cells as in panel **(B)**. **(F)** Graphic representation of flow cytometric values of cell death in A375, KO-A375 and rescued KO-A375 melanoma cell cultures with 100 ng/ml TRAIL (*N* = 3). **(G)** Western blot assay of acEP300, acetyl-lysine, YY1 and DR5 levels in A375, KO-A375 and FKBP51-rescued KO-A375 cells. *p < 0.05, **p < 0.01, ***p < 0.001, ****p < 0.0001.

To strengthen the role of FKBP51 in the regulation of DR5 expression, we generated stable FKBP51-KO with CRISPR/Cas9 technology from both SAN and A375 melanoma cell lines. Western blots from the KO cell lines confirmed that DR5 was negatively regulated by FKBP51 (Figure 5A). In accordance with this finding, both KO cell lines showed an increased sensitivity to TRAIL when compared with control cells (ctrl, carrying the puromycin resistance only) (Figures 5B,C). We then performed FKBP51 rescue of A375-KO. Both flow cytometry (Figure 5D) and RT-qPCR (Figure 5E) showed that DR5 levels decreased when ectopic FKBP51 was transfected in KO. Assessment of cell death showed that rescued KO were insensitive to TRAIL (Figure 5F). We then obtained protein extracts from A375ctrl, A375-KO, and rescued A375KO. Western blot confirmed the effect of ectopic FKBP51 on DR5 and showed that it also reversed the acetylation pattern of KO. Moreover, the level of ac-EP300, the acetylase primarily involved in YY1 acetylation, was also regulated in a similar fashion (Figure 5G).

## Discussion

TRAIL expression by immune cells is a means by which immune cells induce apoptosis of tumor cells (de Looff et al., 2019). TRAIL plays an essential role in CD8 and NK cell-mediated mechanisms of tumor elimination (Takeda et al., 2001, 2002). The tumor microenvironment plays an important role in modulating the efficacy of both the endogenous TRAIL/TRAIL-R system, used by immune cells, as well as of exogenously administrated therapeutic TRAIL receptor agonists (de Looff et al., 2019).

Epigenetic modifications are among the leading causes of TRAIL resistance of cancer cells (Huerta-Yepez et al., 2004). As known, DNA methylation and post-translational modifications of histones profoundly affect gene expression in cancer, driving to uncontrolled proliferation and resistance to cell death (Kanwal and Gupta, 2012). Among the epigenetic modifiers, HDAC inhibitors are considered promising anti-cancer therapeutics (Eckschlager et al., 2017). These agents contrast the highly condensed heterochromatin and non-active transcription status, which relies on histone deacetylation (Passaro and Testa, 2018; Lee et al., 2020). In general, they allow chromatin relaxation and gene expression. HDAC inhibitors were shown to sensitize to TRAIL-induced cell death, coordinating the expression levels of pro-apoptotic and anti-apoptotic proteins (Jazirehi et al., 2014; Perla et al., 2020).

The present study corroborates previous findings of the TRAIL pathway’s positive regulation by HDAC inhibitors in melanoma. Still, it sheds light on an epigenetic mechanism controlled by the immunophilin FKBP51 that TSA contrasts. DR5 expression is regulated at the transcriptional level by YY1. This transcription factor interacts with histone acetyltransferase EP300 and histone deacetylases HDAC1, HDAC2, and HDAC3 (Yao et al., 2001). The repressor activity of YY1 is regulated through acetylation by EP300 and through deacetylation by HDACs (Yao et al., 2001). Huerta-Yepez et al. first showed the direct role of YY1 in DR5 repression; YY1siRNA produced upregulation of DR5 expression and sensitization to TRAIL-induced cell death (Huerta-Yepez et al., 2004). YY1 represses transcription using multiple mechanisms (Elmallah and Micheau, 2019). These mechanisms frequently involve the competition of YY1 with activating factors in overlapping binding sites, thereby decreasing the promoter activity (Elmallah and Micheau, 2019). Accordingly, we found that FKBP51 silencing reduced YY1 binding to the DR5 promoter, along with the expression level of acetyl-YY1.

A general increase of HDACs (as by the proteome assay) is confirmed by the reduced amount of acetylated protein in FKBP51 silenced-melanoma cells (as by Western blot). The observation that TSA contrasted such an effect and restored acetyl-YY1 levels in silenced cells suggests TSA recovers the repressor function of YY1. The strict association of DR5 expression with FKBP51 level was reinforced by rescue experiments in Crispr/Cas9 FKBP51-KO. Intriguingly, the rescued-KO cells appeared to be even insensitive to TRAIL not with standing they expressed DR5. This result could be explained in view of NF-κB mediated resistance stimulated by FKBP51 overexpression (Romano et al., 2015). However, other mechanisms including the effect of an increased expression of the decoy receptor TNFRS10c could not be excluded. Finally, the finding of reduced acEP300 levels in KO and the restoring effect of ectopic FKBP51 suggest a role for FKBP51 as an epigenetic modifier, in accordance with a previous study (Romano et al., 2013).

The proteome assay showed that FKBP51 modulated several TNF-related receptors and ligands. An increased production of the TRAIL-ligand TNFSF10 along with a decrease of the decoy receptor TNFRSF10c (Pan et al., 1997) suggest that additional factors to DR5 could contribute to the improved apoptosis susceptibility of FKBP51-targeted melanoma cells. Also, according to a recent study, TNFRSF18/GITR enhances T cell-mediated killing of melanoma cells (Guo et al., 2019). The observation that this immune checkpoint molecule (Ronchetti et al., 2004) is apparently upregulated in FKBP51-depleted cells is a further element in support that melanoma microenvironment can benefit from FKBP51 targeting.

Gene expression regulation and changes in protein acetylation levels exerted by FKBP51 reasonably implicate an epigenetic function for this immunophilin. Previous studies have involved FKBP51 in the reprogramming of the melanoma cell associated with stemness and EMT (Romano et al., 2013). The finding that FKBP51 can interact with p300 and influences the binding of such a general transcriptional coactivator to certain promoters (Romano et al., 2013) supports the involvement of this immunophilin in chromatin modification. However, its exact role in melanoma epigenetics deserves to be investigated.

## Conclusion

**FIGURE 6:**
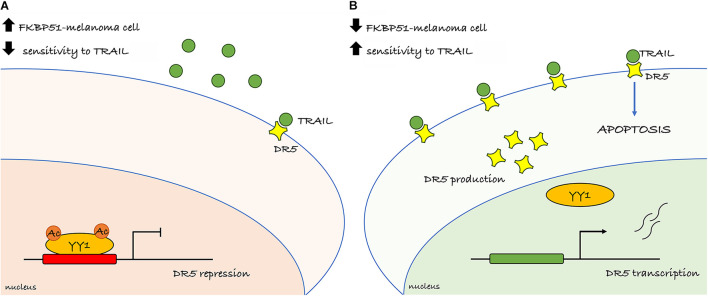
Overview of the primary findings. **(A)** Melanoma cell endowed with high FKBP51 levels presents YY1 in its acetylated form and able to repress the transcription of the DR5 (TRAILR2) and conferring resistance to TRAIL. **(B)** Depletion of FKBP51 impairs the acetylation status of YY1 and interferes with its binding on the DR5 promoter. The lack of the repressor activity of YY1 increases DR5 transcription and sensitizes melanoma cell to TRAIL-induced apoptosis.

We propose that targeting FKBP51 successfully increases TRAIL sensitivity of melanoma. A schematic representation of the primary findings is illustrated in Figure 6. Apoptosis deficiency is a major cause of melanoma therapy failure (Trivedi and Mishra, 2015). Overcoming TRAIL resistance in melanoma may potentiate the tumoricidal action of the immune system and virtually act in concert with checkpoint inhibitor-targeted immunotherapy. It is noticeable that deregulation of TRAIL-R intracellular signaling, primarily due to aberrant NF-κB activation, often deviates signal from cell death to cell survival (Snajdauf et al., 2021). The well-known FKBP51 role in supporting NF-κB activation (Romano et al., 2015) makes this immunophilin an ideal target to restore TRAIL sensitivity of melanoma. Further research is warranted to determine *in vivo* sensitivity to TRAIL of FKBP51-targeted melanoma cells.

## Data Availability Statement

The original contributions presented in the study are included in the article/Supplementary Material, further inquiries can be directed to the corresponding authors.

## Author Contributions

SR and MR conceived the project, designed the experimental plan, and analyzed and interpreted data. MR wrote the manuscript. SR contributed to the manuscript writing. MT and SR performed most of the experimental work. MT generated the CRISPR-Cas9 KO. EC performed the ChIP and protein immunoprecipitation experiments. MR, SR, and RM performed the protein array. RM analyzed the data of the protein array. RP contributed to preparing cells for protein array. All authors discussed the results and commented on the manuscript.

## Conflict of Interest

The authors declare that the research was conducted in the absence of any commercial or financial relationships that could be construed as a potential conflict of interest.

## Publisher’s Note

All claims expressed in this article are solely those of the authors and do not necessarily represent those of their affiliated organizations, or those of the publisher, the editors and the reviewers. Any product that may be evaluated in this article, or claim that may be made by its manufacturer, is not guaranteed or endorsed by the publisher.

## Footnotes

1
https://www.proteinatlas.org/ENSG00000120889-TNFRSF10B/pathology/melanoma#Quantity

2
http://www.sigmaaldrich.com/life-science/cell-biology/protein-arrays/antibody-arrays/725-antibody-microarray.html#sthash.F1ZTO2Zs.dpuf
