# Supplementary material for: FKBP51 Affects TNF-Related Apoptosis Inducing Ligand Response in Melanoma
Source: Front Cell Dev Biol. 2021 Sep 13;9:718947. doi: 10.3389/fcell.2021.718947 (PMC8473884; doi:10.3389/fcell.2021.718947)
Supplement: Supplementary file 1 [file Data_Sheet_1.PDF]

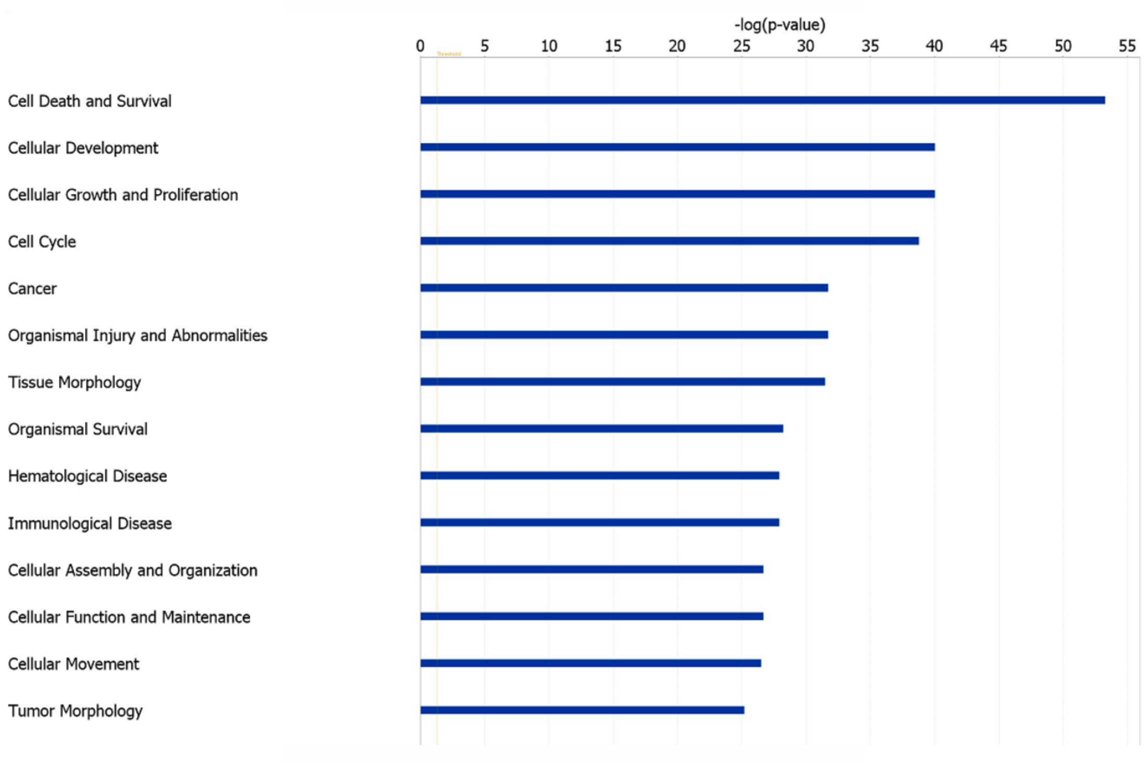

Fig S1. Top categories of diseases and functions generated by IPA program analysis of differentially expressed proteins, in FKBP51 silenced melanoma cells. Categories are ranked by their −log(P-value).
